# Dual Bcl-2/Bcl-xl inhibition via AZD0466 combines with immune checkpoint blockade to enhance anti-tumour activity

**DOI:** 10.1038/s41419-025-08354-w

**Published:** 2026-01-06

**Authors:** Dane M. Newman, Courtney L. Andersen, Leonie A. Cluse, Andrea Newbold, Peter Fraser, Benjamin W. C. Legg, Luyao Kevin Xu, Deanna A. Mele, Ricky W. Johnstone

**Affiliations:** 1 The Sir Peter MacCallum Department of Oncology, University of Melbourne, Parkville, VIC Australia; 2 Gene Regulation Laboratory, Peter MacCallum Cancer Centre, Grattan Street, 3000 Melbourne, Australia; 3 Oncology R&D, AstraZeneca, Waltham, MA USA

**Keywords:** Tumour immunology, Tumour immunology

## Abstract

Small molecule inhibitors designed to specifically target oncogenic proteins have demonstrated potent anti-tumour activities due to direct effects on tumour cells survival and/or proliferation. However, the effects of these compounds on normal cells, specifically immune cells and their potential to impede or enhance anti-cancer immunotherapies has yet to be fully explored. Using an in vitro co-culture system to assess CD8+ T cell killing of tumour cells, we identified compounds that inhibit Bcl-2 and Bcl-xl as agents that can induce tumour cell death without impacting the differentiation or function of anti-tumour T cells. Accordingly, in vivo treatment of mice bearing solid tumours with a combination of the Bcl-2/Bcl-xl inhibitor AZD0466 and anti-PD-L1 immunotherapy resulted in enhanced anti-tumour effects and improved survival compared to equivalent monotherapies.

## Introduction

Small molecules that directly target the oncogenic dependencies of tumour cells have been an important advance for the treatment of cancer [1, 2]. While the preclinical progression of new oncology agents has predominately focussed on the cell-intrinsic effects on tumours, there is increasing recognition of the impacts that these compounds may have on immune cells and broader antitumour immune responses [3]. Therefore, maximising the therapeutic potential of small molecule inhibitors requires consideration of the functional impacts on tumour and immune cells concurrently.

The recent advent of immunotherapies such as immune checkpoint blockade (ICB) has revolutionised the treatment of many cancer types [4]. ICB employs targeted antibodies to disrupt the binding interactions between checkpoint proteins on the cell surface of cytotoxic T cells (e.g. PD-1) and their associated ligands expressed on the tumour cell surface (e.g. PD-L1), leading to a reinvigoration of the anti-tumour T cell response and in many cases, clinical remission [5]. However, absent or incomplete ICB responses in some cancer types or within patient cohorts has prompted investigation into combining immunotherapies with other established anticancer regimes, including the use of small molecule inhibitors [6–8]. It is therefore crucial to identify agents that can support or augment tumour-specific T cell killing, particularly in the context of ICB therapy.

Here we used an antigen-specific T cell killing screen to assess the immune-modulatory activity of a range of clinically relevant anti-cancer compounds. These in vitro screens identified a role for AZD4320, a dual Bcl-2/Bcl-xl inhibitor, in enhancing CD8+ T cell killing of tumours, with no observable impacts on T cell differentiation or function. Moreover, treatment of tumour-bearing mice with AZD0466, a drug-dendrimer conjugate of AZD4320, combined with anti-PDL1 therapy derived superior anti-tumour activity in comparison to equivalent monotherapies. Our data provide strong impetus for additional studies combining dual Bcl-2/Bcl-xl inhibition with ICB and other immune targeting therapies.

## Results

### CD8+ T cell killing of tumours is bolstered by Bcl-2/Bcl-xl inhibition

**Fig. 1:**
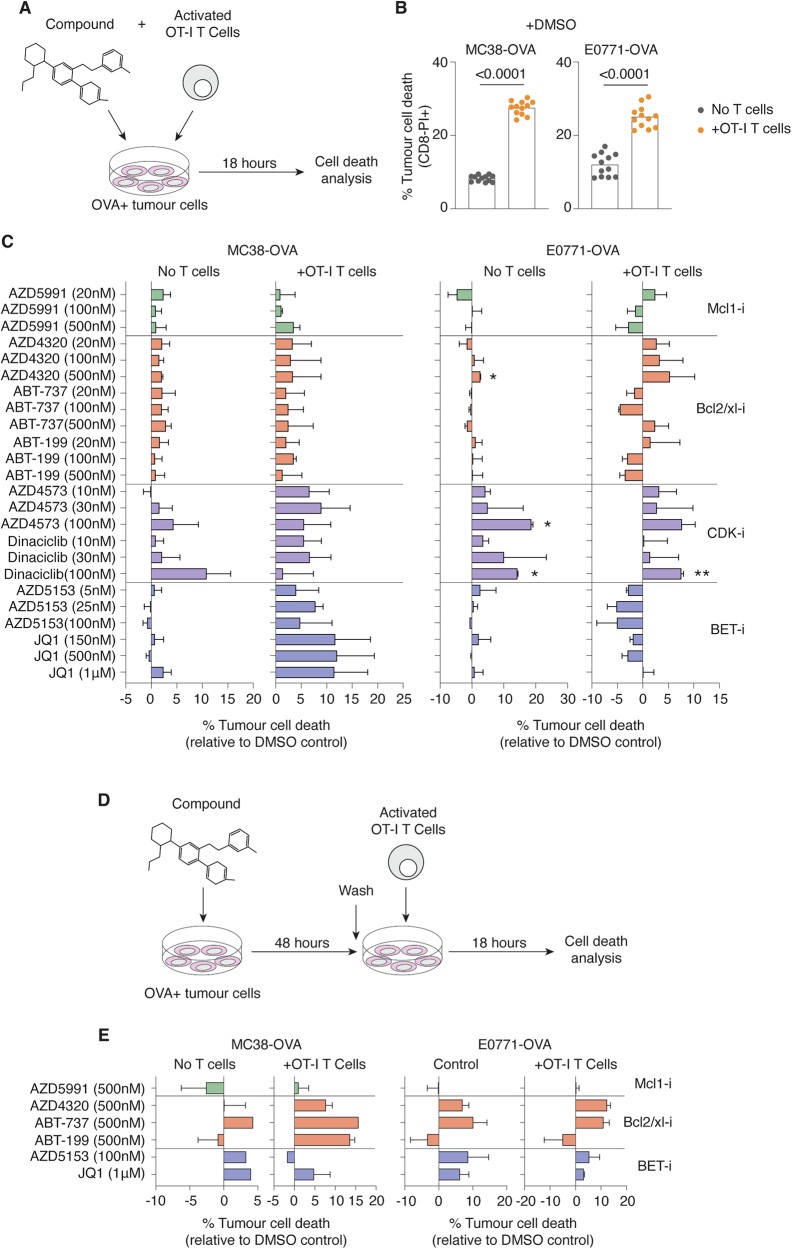
Identifying small molecule inhibitors that promote T cell killing of tumours in vitro. **A** Experimental scheme. OVA-expressing MC38 or E0771 tumour cells were combined with activated OT-I CD8+ T cells and compound and co-cultured for 18 h prior to cell death analysis. **B** Comparison of tumour cell death percentages with or without addition of OT-I T cells. (**C**) Relative differences in tumour cell death (compared to DMSO control) of cultures treated with various small molecule inhibitors. **D** Experimental scheme. OVA-expressing tumour cells were pre-treated with compound for 48 h prior to co-culture with activated OT-I T CD8+ T cells. **E** Differences in percent cell death of pre-treated tumour cells relative to DMSO control. Data are representative of two independent experiments. Error bars represent ±SEM for all figures. *p < 0.05; **p < 0.01.

To identify small molecule anti-cancer agents that have potential to synergise with ICB therapies, an in vitro co-culture system was designed to simultaneously assess the impact of compounds on CD8+ T cell-mediated killing of tumour cells. Effector T cells from OT-I transgenic mice, which recognise ovalbumin (OVA)-peptide 257-264 presented in the context of H2K^b^, were activated in vitro and then combined with OVA-expressing mouse colon adenocarcinoma (MC38-OVA) or mammary cancer (E0771-OVA) cell lines in the presence/absence of compounds (Fig. 1A). Overnight co-culture of OT-I T cells and OVA + MC38 and E0771 tumour cells led to a 3-fold and 2-fold increase in tumour cell death respectively (Fig. 1B). Additionally, comparison of OVA-expressing or parental MC38 cell lines verified that OT-I T cell killing was specific to OVA-expressing tumours (Supplementary Fig. 1A).

Tumour cells co-cultured with OT-I T cells and a panel of small molecule inhibitors resulted in varying levels of cell death (Fig. 1C). Bromodomain (BET) inhibitors JQ1 and AZD5153 enhanced OT-I T-mediated killing of MC38-OVA tumours cells (~10% increase) but were largely non-toxic in the absence of T cells, an observation in line with previous reports of enhanced anti-tumour immunity following BETi treatment [9]. However, T cell killing of E0771-OVA tumour cells appeared unaffected or was even impeded by BET inhibition, suggesting a cell-specific mechanism of action. In contrast, the dual Bcl-2/Bcl-xl inhibitor AZD4320 sustained baseline T cell killing and modestly enhanced overall cell death of both MC38-OVA and E0771-OVA cell lines (Fig. 1C). These observations were further verified to occur in an antigen-specific manner (Supplementary Fig. 1B). The Mcl-1 inhibitor AZD5991 exerted limited influence on tumour cell death, while co-cultures treated with CDK inhibitors AZD4573 and Dinaciclib exhibited high levels of toxicity in the absence of T cells (Fig. 1C).

To further assess the potential of small molecules to sensitise tumour cells to immune killing, a modified co-culture system was designed whereby MC38-OVA or E0771-OVA cells were pre-treated with compound for 48 h prior to combining with OT-I T cells in co-culture (Fig. 1D). The CDK inhibitors AZD4573 and Dinaciclib induced high levels of tumour cell death during pre-treatment and were excluded from analysis (data not shown). Pre-treatment of tumour cells with JQ1 and AZD5153 increased tumour cell killing in control cultures via direct toxicity, but addition of OT-I T cells provided limited additive potency. Notably, pre-treatment of MC38-OVA cells with Bcl-2 inhibitor ABT-199 and dual Bcl-2/Bcl-xl inhibitors ABT-737 and AZD4320 appeared to sensitise tumours to immune killing, with tumour cell death noticeably increased following T cell co-culture (Fig. 1D). Increased E0771-OVA cell death was also observed in both control- and co-cultures following ABT-737 and AZD4320 pre-treatment. In contrast, exposure of tumour cells to the Mcl1 inhibitor AZD5991, had limited effects on baseline cell death. Taken together, these co-culture screening assays revealed a capacity for inhibitors of Bcl2 and Bcl2/Bcl-xl to enhance CD8+ T cell killing of tumours.

### Differentiation and function of CD8+ T cells is unaffected by Bcl-2/Bcl-xl inhibition

**Fig. 2:**
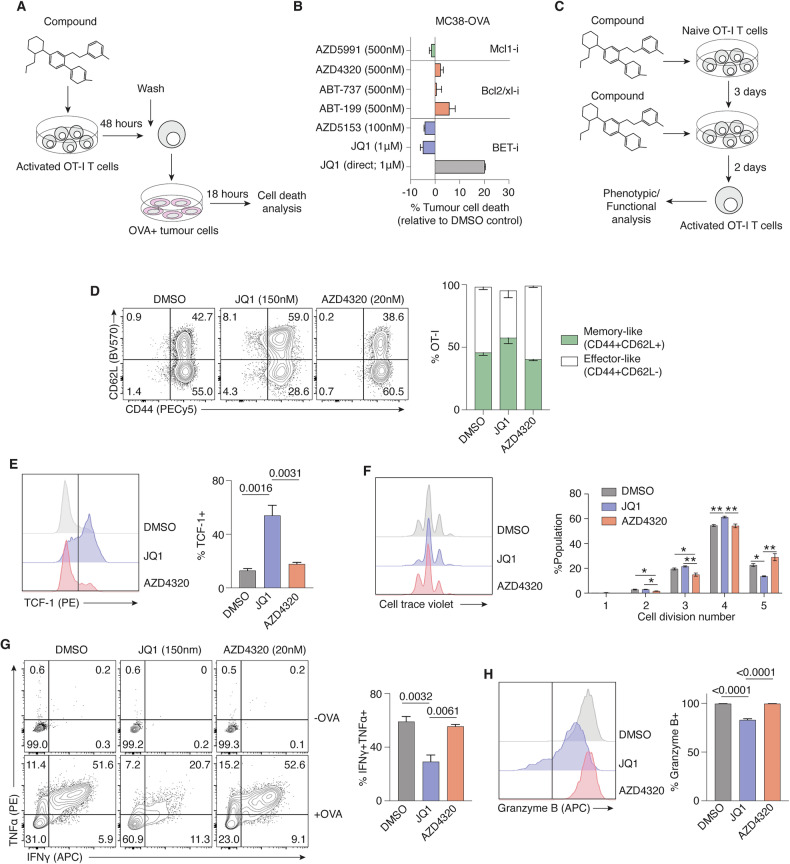
In vitro CD8+ T cell differentiation and function unimpeded by Bcl-2/Bcl-xl inhibition. **A** Experimental scheme. OT-I CD8+ T cells were activated and pre-treated with small molecule inhibitors for 48 h prior to combining with OVA-expressing tumour cells for overnight co-culture. (**B**) Percent tumour cell death following co-culture with OT-I T cells pre-treated with various compounds. Direct treatment of co-culture with JQ1 (JQ1 direct) was included as a positive control. **C** Experimental scheme. Naïve OT-I T cells were activated and cultured in the presence of small molecule inhibitors for a total of five days prior to analysis. **D** Representative flow plots of CD62L versus CD44 expression of OT-I T cells treated with JQ1, AZD4320 or DMSO control. Graph on right shows percent of memory-like (CD62L+CD44+) or effector-like T cells (CD62L-CD44+) across treated OT-I populations. **E** Flow histogram depicting intracellular expression of TCF-1 (left) and percent TCF-1+ cells (right) of treated OT-I T cell populations. **F** Representative flow plots of cell trace violet (CTV)-stained OT-I T cell populations across treated populations (left) and percent of total OTI population that have undergone 1-5 cell divisions (right). *p < 0.05; **p < 0.01 **G** Representative flow plots of intracellular TNFα and IFNγ expression of treated OT-I T cell populations with or without OVA-restimulation. Graph on right shows percent of OT-I T cell populations expressing both TNFα and IFNγ. **H** Flow histogram depicting intracellular expression of Granzyme B (left) and percent Granzyme-B+ cells (right) of treated OT-I T cell populations. Data are representative of 2 or 3 independent experiments. Error bars represent ±SEM for all figures. Exact significant (*p*) values are shown.

The developmental transition of CD8+ T cells from a naïve state to activated effector is a finely tuned process and perturbations to these regulatory networks can greatly alter T cell differentiation and function [10, 11]. To compare the effect of small molecule inhibitors on T cell development and anti-tumour activity, the co-culture screen was adapted to enable pre-treatment of OT-I T cells with compounds prior to combining with target tumour cells (Fig. 2A). As expected, direct JQ1 treatment of both OT-I T cells and target cells in co-culture increased MC38-OVA cell death, while pre-treatment of T cells with JQ1 or alternative BET inhibitor AZD5153 resulted in negligible, or slightly reduced killing of target cells relative to baseline (Fig. 2B). Pre-treatment of OT-I T cells with Bcl-2 family inhibitors ABT-199, ABT-737 and AZD4320 or AZD5991 also had minimal impacts on subsequent anti-tumour activity, suggesting that the functions of OT-I T cells were largely unaffected by these conditioning regimes.

Given that relative efficacy of T cell killing of tumours appeared augmented by dual Bcl-2/Bcl-xl inhibition (Fig. 1C, E), the effects of AZD4320 on T cell differentiation and function were investigated. OT-I T cells were cultured with DMSO, AZD4320 or JQ1 (a known modulator of T cell differentiation [9, 12, 13]), for five days beginning from the point of initial naïve T cell activation (Fig. 2C). Following activation, T cells upregulate the expression of CD44, a marker of antigen-experience, and further undergo a differentiation process that culminate in divergent cell states including a CD62L-low effector-like phenotype, and a CD62L-high memory-like phenotype (Fig. 2D). As previously described [13], JQ1 treatment of OT-I T cells resulted in a significant increase in the proportion of CD62L+ memory-like T cells relative to DMSO control T cells (Fig. 2D). Moreover, JQ1-treated OT-I T cells expressed significantly higher intracellular levels of the transcription factor TCF-1, a key marker of T cell memory (Fig. 2E). In contrast, treatment of differentiating OT-I T cells with AZD4320 largely phenocopied control T cells (Fig. 2D–E), suggesting minimal impact of Bcl-2/Bcl-xl inhibition on early T cell differentiation into CD62L-TCF-1- effector cells. Cell proliferation, as indicated by cell trace violet (CTV) dilution (Fig. 2F), also appeared largely unaltered by JQ1 or AZD4320 treatment. Similarly, human CD8+ or CD4 + T cells activated and expanded in the presence of AZD4320 for three days exhibited only modest dose-dependent reductions in T cell number, with naïve CD8+ T cells exhibiting higher sensitivity Bcl-2/Bcl-xl inhibition (Supplementary Fig. 2), as has been previously described following Bcl-2 inhibition [14].

To examine T cell effector functions following Bcl-2/Bcl-xl inhibition, pre-treated OT-I T cells were restimulated with OVA peptide and evaluated for the production of intracellular cytokines IFN-γ and TNFα (Fig. 2G). Following antigen recognition, DMSO-treated OT-I T cells exhibited a higher proportion (59.2%) of T cells expressing both IFN-γ and TNFα, whereas T cells pre-conditioned in JQ1 exhibited decreased polyfunctionality (29.2% IFN-γ + TNFα+), an observation in line with previous reports of lower cytotoxic function in memory-like T cells [15]. In contrast, pre-treatment of OT-I T cells with AZD4320 did not greatly alter effector cytokine production (55.6% IFN-γ + TNFα+). Intracellular granzyme B expression of OT-I T cells was also partly suppressed by JQ1 treatment (82.9% granzyme B + ) but unaffected by AZD4320 treatment (99.7%) relative to DMSO control (99.7%; Fig. 2H), further supporting the notion that Bcl-2/Bcl-xl inhibition had limited impact on overall CD8+ T cell differentiation and function.

### Dual Bcl-2/Bcl-xl inhibition enhances immune checkpoint therapy in vivo

**Fig. 3:**
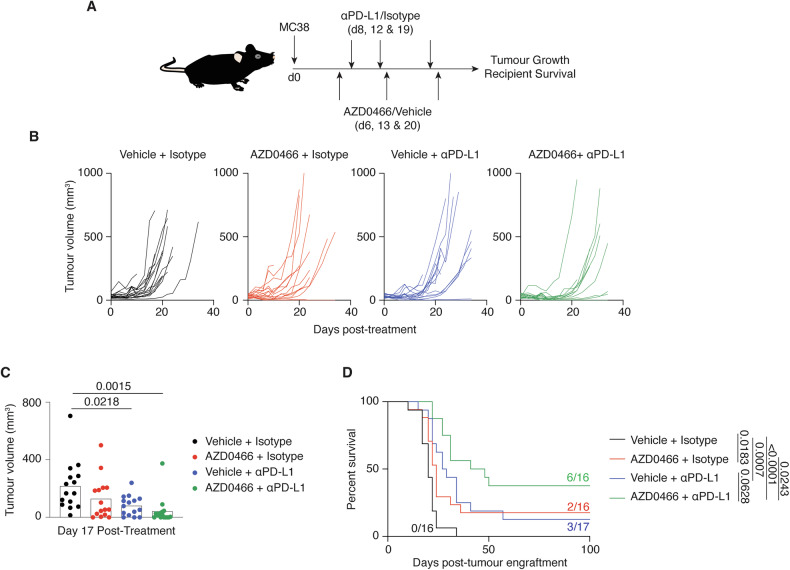
Dual Bcl-2/Bcl-xl inhibition combines with immune checkpoint therapy to enhance anti-tumour effect in vivo. **A** Experimental scheme. C57BL/6 mice with established subcutaneous MC38 tumours were treated with AZD0466, αPD-L1 or a combination thereof from 6–20 days after tumour engraftment. **B** Growth of individual MC38 tumours in mice under each treatment condition and **C** tumour size on day 17 post engraftment. **D** Kaplan–Meier curve showing survival of treated mice. Proportion of recipients that cleared tumours are indicated. Data are representative of two independent experiments. Exact significant (*p*) values are shown.

To assess the anti-tumour activity of Bcl-2/Bcl-xl inhibition in vivo, an AZD-4320 dendrimer conjugate (AZD0466) was employed in order to limit toxicity and maximise therapeutic efficacy [16]. MC38 tumour cells were engrafted subcutaneously into syngeneic mouse recipients and subsequently treated with an immune checkpoint antibody targeting PD-L1 (αPD-L1), AZD0466, or a combination of both agents (Fig. 3A). Seventeen days following treatment commencement, single agent therapy using AZD0466 or αPD-L1 had decreased the growth of tumours by 41% and 63% respectively compared to vehicle-treated mice, while combination therapy resulted in more pronounced inhibition of tumour growth (81%) (Fig. 3B, C). The superior anti-tumour activity of combined AZD0466 + αPD-L1 treatment also led to significantly prolonged survival of recipients with 6/16 recipients (37%) having no observable tumour at 100 days post-engraftment (Fig. 3D). In contrast, single agent treatment with AZD0466 or αPD-L1 resulted in 3/17 (17%) and 2/16 (12%) survival at 100 days, while no mice in the control group survived beyond day 34 post-engraftment. A similar, albeit less pronounced combinatorial effect of AZD0466 and αPD-L1 was observed in an AT3-OVA mouse solid tumour model (Supplementary Fig. 3). These data further indicate the immune-supportive attributes of Bcl2/Bcl-xl inhibition and reinforced the observations made from in vitro screens.

### Tumour infiltrates and immune memory unaffected by AZD0466 therapy

**Fig. 4:**
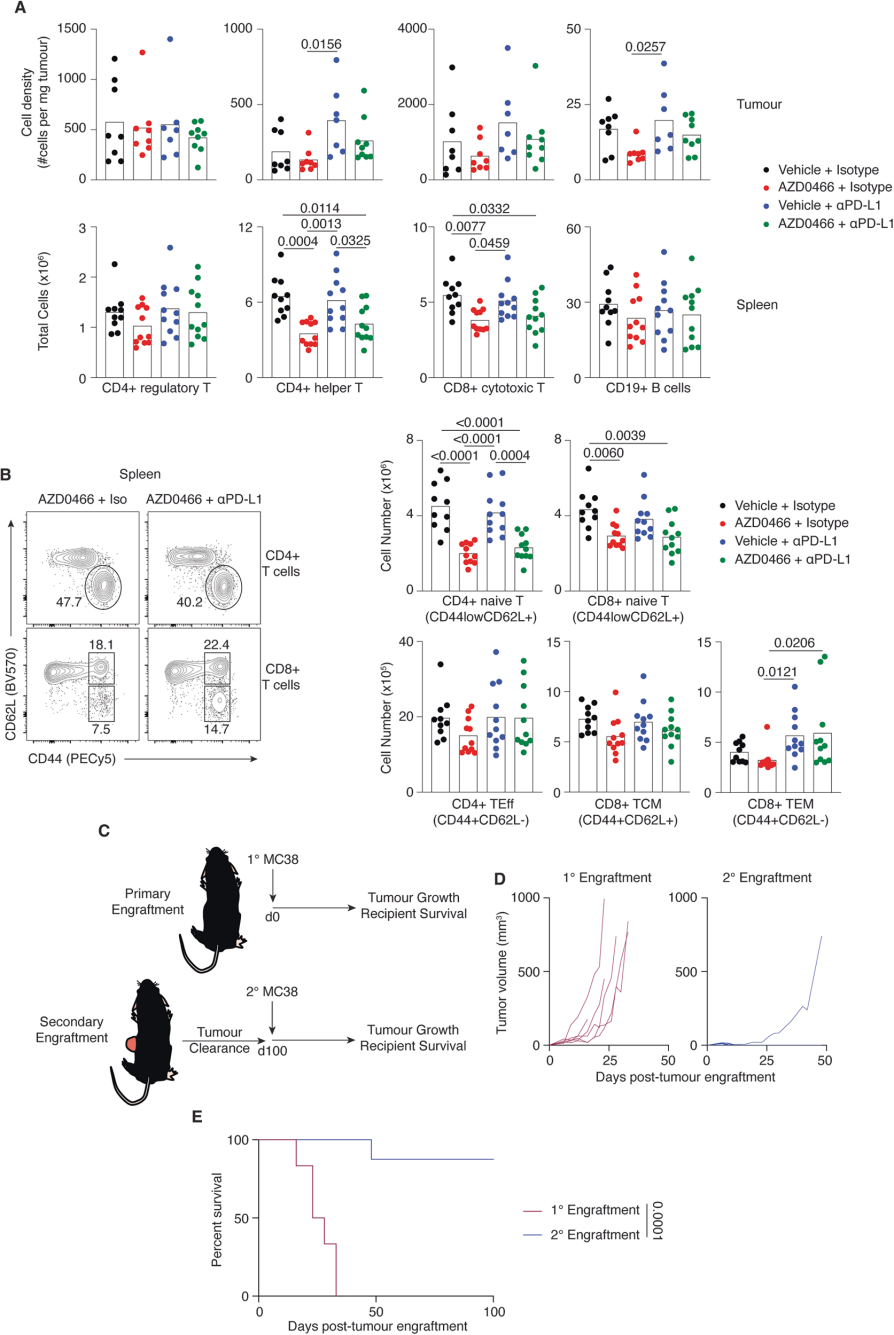
Robust tumour-infiltrating immune landscape and immune memory following Bcl-2/Bcl-xl treatment. **A** Cell density (tumour, top row) and total numbers (spleen, bottom row) of T cell subsets and B cells from mice at 17 days following commencement of treatment. **B** Representative flow plots of CD62L versus CD44 expression of CD4+ and CD8+ T cell subsets from the spleen. Graphs on right shows total numbers of naïve CD4+ or CD8+ T cells (CD62L+CD44low), CD4+ effectors (T_Eff_; CD62L-CD44+), CD8+ central memory (T_CM_; CD62L+CD44+) and CD8+ effector memory (T_EM_; CD62L-CD44+) from spleens of treated mice. **C** Experiment schematic. Naïve mice or mice that had previously cleared tumours following AZD0466-treatment mice were engrafted, or re-engrafted, with subcutaneous MC38 tumours. **D** Growth of individual MC38 tumours in mice that received primary or secondary engraftments. **E** Kaplan–Meier curve depicting survival of mice from each group. Data are representative of two independent experiments. Exact significant (*p*) values are shown.

Given a potential immune enhancing effect of AZD0466 treatment, flow-cytometric analysis of tumour infiltrates from both MC38 and AT3-OVA tumours were performed ex vivo at 17 days following treatment commencement (see gating strategy Supplementary Fig. 4). Relative lymphocyte densities (FoxP3+ regulatory T cells, FoxP3- CD4+ T helper cells, CD8+ T cells and CD19 + B cells) within MC38 or AT3-OVA tumours appeared largely unaltered by Bcl-2/Bcl-xl inhibition, with or without αPD-L1 therapy (Fig. 4A; Supplementary Fig. 5A). However, total numbers of CD4+ helper T cells and CD8+ cytotoxic T cells in the spleen were significantly lower in AZD0466 treated MC38-bearing mice (Fig. 4A).

To examine the deficit in splenic T cells in greater detail, we compared treatment-related differences of naïve (CD44_low_CD62L+) CD4+ and CD8+ T cells, as well as effector CD4 + CD44 + CD62L- T cells (CD4+ T_Eff_), CD44 + CD62L + CD8+ central memory (CD8+ T_CM_) and CD44+CD62L-CD8+ effector memory (CD8+ T_EM_) T cell populations (Fig. 4B). The observed decrease in overall T cell numbers in the spleens of AZD0466 treated mice appeared largely due to smaller populations of naïve CD4+ and CD8+ T cells, whereas the numbers of CD4+ T_Eff_ and CD8+ T_CM_ and T_EM_ appeared relatively normal, particularly in mice that received combination therapy. Indeed, an expanded number of cytotoxic CD8+ T_EM_ in the spleen following αPD-L1 treatment was also observed in mice receiving combination therapy, suggesting the activation and subsequent ICB-related reinvigoration of effector T cells was maintained by AZD0466 treatment.

The anti-tumour efficacy of ICB treatment is largely dependent upon a distinct progenitor CD8+ T cell subset, referred to as ‘precursor of exhausted T cells’ (TPEX) [17] that become activated in the tumour draining lymph node (TdLN) and give rise to effector and exhausted T cell populations within the tumour [18, 19]. To better understand the immune-related effects of combined AZD0466/αPD-L1 therapy, we compared treatment-related differences in CD8+ T cell number and function of PD-1+TCF-1+Tim3- TPEX and their more terminally differentiated progeny PD-1+TCF-1-Tim3+ ‘exhausted’ T cells (TEX) in both the tumour and TdLN of AT3-OVA bearing mice. Populations of TdLN-resident TPEX appeared modestly reduced in mice treated with AZD0466 monotherapy, although this difference was largely overcome in mice receiving combined AZD0466/αPD-L1 treatment. Additionally, overall densities of TPEX and their TEX progeny within the tumour were also broadly unaffected by Bcl-2/Bcl-xl inhibition (Supplementary Fig. 5B), while activation/proliferation potential (as indicated by Ki67 expression) and cytotoxic capacity (granzyme B expression) of both TPEX and TEX were also unaffected by AZD0466 treatment (Supplementary Fig. 5C). These data further indicate a negligible impact of Bcl-2/Bcl-xl inhibition on ICB-mediated T cell responses.

Myeloid cell densities in MC38 tumours showed little change following treatment, although the relative densities of neutrophils were modestly increased following AZD0466 monotherapy (Supplementary Fig. 6). Similar observations were made in the spleen, where total numbers of myeloid cell types were broadly comparable regardless of treatment (Supplementary Fig. 6).

Lymphocytopenia and thrombocytopenia are known side effects of Bcl-2 and Bcl-xl inhibition [20, 21], although AZD4320/AZD0466 have been reported to overcome some dose-limiting toxicities [16, 22]. White blood cell and platelet numbers were therefore examined in the peripheral blood of MC38-tumour bearing mice over the treatment period (Supplementary Fig. 7). Treatment-related decreases in platelet numbers in the blood were observed after 14 days of AZD0466-treatment, causing notable thrombocytopenia after 21 days (Supplementary Fig. 7A), thus confirming an on-target effect on Bcl-xl inhibition, but perhaps indicating a cumulative effect of multiple AZD0466 doses on platelet survival in this particular mouse model. White blood cell counts were significantly lower in the blood following the first week of AZD0466 treatment, but stabilised thereafter (Supplementary Fig. 7B). These reduced blood counts are somewhat at odds with the relatively normal white blood cell counts, particularly CD19 + B cells, in the spleen of AZD0466-treated mice (Fig. 4A). The specific factors underpinning these anatomical discrepancies are unknown and require additional investigation.

To further examine the integrity of the immune landscape following treatment, an additional tumour challenge experiment was designed whereby mice that had successfully cleared tumours following AZD0466 treatment (Fig. 3D; *n* = 8) were re-engrafted with new MC38 tumours (Fig. 4C). Vehicle-treated groups were not included in this assay given the low number of mice that successfully cleared tumours (*n* = 3), thus limiting a robust comparison. Tumour growth and recipient survival was then compared in parallel with an equivalent cohort of wild type (i.e. tumour inexperienced) mice receiving primary engraftment of MC38 tumours. All mice receiving primary engraftment demonstrated exponential tumour growth from as day 9, whereas only one mouse from the secondary engraftment cohort developed a palpable tumour (Fig. 4D). Exponential development of this tumour was also protracted (~day 25) relative to those in the primary engraftment group, suggesting an early period of immune control. No mice receiving primary tumours survived beyond day 33, whereas 6/7 mice (86%) that received secondary engraftment remained viable with no observable tumour at 100 days (Fig. 4D), indicating the formation of robust immune memory in mice previously treated with AZD0466 and αPD-L1.

Collectively, these data indicated that dual Bcl-2/Bcl-xl inhibition supports a robust anti-tumour immune response and significantly improves overall therapeutic outcomes when combined with ICB.

## Discussion

Molecular targeted therapy and immunotherapy represent the two most recent pillars of cancer treatment, yet despite compelling clinical outcomes in some cancer types, neither has proven to be broadly effective as monotherapies. Therefore, the design of rational combination regimens that target both the molecular events that underpin tumour cell proliferation and/or survival while also stimulating the anti-tumour immune response offers great promise in addressing the shortcomings of single-target therapies. By comparing the modulatory effects of a panel of small molecule inhibitors on tumour and immune cells concurrently, our data reveal a previously unrecognised ability of the Bcl-2/Bcl-xl inhibitor AZD4320 (or AZD0466) in enhancing anti-tumour immune activity and bolstering the response to ICB in vitro and in vivo.

Molecular therapies designed to inhibit the anti-apoptotic protein Bcl-2 have experienced remarkable clinical successes, leading to FDA approval of the BH3-mimetic ABT-199 (Venetoclax) for treatment of various haematological cancers [23]. In contrast, clinical progression of dual Bcl-2/Bcl-xl inhibitors such as Navitoclax (ABT-263) have been hampered by dose-limiting toxicity and thrombocytopenia [24]. The recent development of AZD0466—a drug-dendrimer conjugate to the Bcl-2/Bcl-xl inhibitor AZD4320, designed for intermittent intravenous administration—has been proposed as a less toxic alternative to sustained oral delivery of Navitoclax, while still maintaining efficacy [16, 22]. However, the indirect impacts of AZD0466 on immune cells and anti-tumour immunity have yet to be established.

Immune cells have important, yet varying dependencies on Bcl-2 protein family members [25] and targeted inhibition of Bcl-2/Bcl-xl (ABT-737/Navitoclax) or Bcl-2 alone (Venetoclax) have been reported to induce differing levels of toxicity depending on cell type or differentiation status [14, 21, 26–28]. Nevertheless, Kohlhapp et al. [29] demonstrated that syngeneic mouse solid tumour models treated with a combination of Venetoclax and αPD-1 or αPD-L1 therapy resulted in increased numbers of tumour-infiltrating CD8+ effector T cells and superior T cell-mediated tumour regression. Furthermore, a recent study by Teh et al. [30] observed that cancer patients treated with Venetoclax maintained healthy and functional populations of circulating T and NK cells, thus substantiating the clinical potential of combining ICB with Bcl-2 inhibition. Notably, the absence of treatment-related effector CD8+ T cell toxicity observed in the Kohlhapp et al. study was proposed to be due to an upregulation of Bcl-xl in these cells, thus compensating for the loss of Bcl-2 and thereby preventing T cell apoptosis [29]. Data from the present study, however, indicated that dual inhibition of Bcl-2 and Bcl-xl exerted minimal impact on effector CD8+ T cells activity and ultimately enhanced the overall therapeutic efficacy of ICB, akin to Venetoclax treatment [29, 31]. Dual antagonism of both Bcl-2 family proteins therefore appears to enhance anti-tumour immunity and remains a viable alternative to Venetoclax-based regimens, especially in situations where Bcl-xl may be important for maintenance of tumour cell viability. Further mechanistic studies are required to understand the cellular and molecular mechanisms that underpin the therapeutic response of combined AZD0466/ICB therapy. Importantly this study, using an unbiased screening approach to identify agents that augment CD8 T cell mediated anti-tumour responses provides the basis for further refinement of AZD0466/ICB therapy and future screens using a more expansive array of small molecules and cytotoxic drugs.

The development of dual Bcl-2/Bcl-xl antagonists promise to address the clinical hurdles associated with Venetoclax-resistant, Bcl-xl dependent tumours. The present study builds upon this notion by revealing a previously unrecognised capacity of AZD0466 as an effective adjuvant for immunotherapy. Given that many clinical trials are currently evaluating Venetoclax and ICB combinations (NCT04277442; NCT05388006; NCT03969446; NCT02846623), our study provides strong rationale for additional enquiry into the clinical benefits of combining dual Bcl-2/Bcl-xl inhibitors with immune-stimulating therapies.

## Materials and methods

### Mice

Wildtype C57BL/6J mice were obtained from the Walter and Eliza Hall Institute, while OT-I mice (JAX: Tg(TcraTcrb)1100Mjb/J) were bred in-house at the Peter MacCallum Cancer Centre. All mice were housed under specific pathogen-free conditions (SPF) and used for experiments at 6 - 10 weeks of age. All animal procedures used in this study were approved by the Peter MacCallum Cancer Centre Animal Ethics Committee.

### Tumour and OTI T cell cultures

Mouse MC38, MC38-OVA, E0771-OVA and AT3-OVA cell lines were maintained at 37°C at 10% CO_2_ in DMEM supplemented with 10% FCS, 2 mmol/l GlutaMax and 50 U/ml penicillin/streptomycin and were tested negative for mycoplasma. For T cell cultures, whole spleens from transgenic OT-I mice were disassociated into a single cell suspension through a 70μM nylon cell strainer and resuspended in RPMI media supplemented with 10% FCS, 2 mmol/l GlutaMax, 50 U/ml penicillin/streptomycin, 1 mmol/l sodium pyruvate, 10 mmol/l HEPES, 1 x nonessential amino acids, and 50 μmol/l 2b-mercaptoethanol. Splenocyte concentration was adjusted to 5–10^4^ naïve OT-I cells/ml and cultured with 20 ng/ml SIINFEKL (OVA) peptide and recombinant human IL-2 (100 IU/ml; Biolegend) for 3 days at 37 °C at 5% CO_2_. Expanded OT-I T cells were passaged into fresh IL-2 containing media and cultured for an additional two days prior to use.

### Cell co-cultures

Parental or OVA-expressing MC38 or E0771 cells were plated at 5 × 10^4^ cells per well of a 96-well plate and allowed to adhere for at least 6 to 8 h. Activated OT-I T cells (described above) were washed in complete DMEM and transferred to the tumour cell plate to obtain an effector:target ratio of 1:8. Co-cultures were subsequently incubated at 37 °C at 10% CO_2_ for 18–20 h prior to analysis. At harvest, supernatant containing dead cells and OT-I T cells was collected from each well before collecting adherent tumour cells using trypsin. Total well contents were washed in flow cytometry buffer (PBS with 2% FCS and 5 mmol/l EDTA) and stained with anti-mouse CD8α (53-6.7; 1:200; Biolegend) on ice for 20 min. Samples were washed again in flow cytometry buffer and 2 μg/ml of PI was added immediately prior to analysis, which was performed on an LSR II flow cytometer (BD Biosciences). OT-I T cells were excluded from subsequent analyses of PI+ cell death by gating out the CD8α + fraction.

### OT-I T cell analyses

For analysis of T cell phenotype, expanded OT-I T cells were washed in flow cytometry buffer and stained with antibodies targeting CD8α (53-6.7; 1:200), TCR.Vα2 (B20.1; 1:200), CD44 (IM7; 1:600) and CD62L (MEL-14; 1:200; all Biolegend) on ice for 20 min. Samples were washed again in flow cytometry buffer and 2 μg/ml of PI was added immediately prior to analysis on an LSR II flow cytometer (BD Biosciences). For intracellular transcription factor analysis, surface-stained OT-I T cells were incubated in fixable viability dye (Zombie Aqua, 1:500; Biolegend), fixed and permeabilized using an intracellular transcription factor staining kit (Ebioscience) before staining with anti-TCF-7/TCF-1 antibody (S33-966, 1:50; BD Bioscience) or anti-Granzyme B (GB12, 1:200; Invitrogen). For tracking of cell proliferation, naïve OTI T cells were isolated (EasySep isolation kit; Stemcell Technologies) and suspended at a density of <2 ×107 cells/ml in PBS + 0.1% bovine serum albumin (BSA) containing 5μM of CellTrace Violet (Molecular Probes; Thermo Fisher Scientific). Cell suspensions were incubated in a 37 °C water bath for 20 min, prior to washing in cold culture media and subsequent activation and culture. For functional T cell analyses, OT-I T cells cultured for five days were washed in supplemented RPMI (as above) and incubated for 5 h at 37 °C at 5% CO_2_ with 20 ng/ml SIINFEKL peptide (Sigma Aldrich) and 0.7 μl/ml Golgi Stop (BD Bioscience), or Golgi Stop alone (unstimulated control). Cells were then washed in flow cytometry buffer, stained in fixable viability dye (as above), fixed and permeabilized using the Cytofix/Cytoperm kit (BD Bioscience), and stained with anti-IFN-γ (XMG1.2, 1:100; BD Bioscience) or TNFα (MP6-XT22, 1:100; Ebioscience). Samples were washed in flow cytometry buffer prior to analysis.

### Human T cell culture and analysis

Naïve human CD3+ T cells were isolated from PBMC using a negative selection kit (StemCell Technologies) and activated with antibodies targeting CD3 (OKT3) and CD28 (CD28.2) and cultured in the presence/absence of AZD4320 for 72 h. Expanded cells were subsequently analysed via flow cytometry using an 8-colour anti-human immunophenotyping kit (REAfinity, Miltenyi Biotec).

### In vivo tumour models

Mice were shaved on the right flank and 1 × 10^6^ MC38-parental cells or 5 × 10^5^ AT3-OVA cells were injected subcutaneously. Tumour-bearing mice were randomised into treatment groups 6 days following tumour inoculation and subsequently treated I.V. with AZD0466 (34 mg/kg) or vehicle (citrate/phosphate buffer pH 5.0 with 5% glucose) on days 6, 13, and 20. Antibodies targeting PD-L1 (10 F.9G2; Bioxcell; 10 mg/kg) or isotype (LTF-2; Bioxcell) were administered I.P. on days 8, 12 and 19. Tumours were measured with a caliper in two dimensions (*a* = length; *b* = width) three times a week and tumour volume (*V*) was calculated using the following equation: *V* = *ab*2/2. A maximal tumour size of 1400mm^3^, as approved by the Peter MacCallum Cancer Centre Animal Ethics Committee, was not exceeded in this study.

### Analysis of immune cells ex vivo

At harvest, tumour-bearing mice were euthanized via CO_2_ asphyxiation and cardiac perfused with 10 ml of cold PBS to remove circulating cells from tumour tissue prior to excision. Whole spleens or tumour-draining lymph nodes were removed, placed in flow cytometry buffer, and disassociated into a single cell suspension through a 70 μM nylon cell strainer. Tumour tissue was removed, weighed, and finely chopped with scissors in 1 ml of RPMI supplemented with 2.5% FCS. Tumour pieces were transferred into 20 ml of pre-warmed RPMI-based digestion solution containing 100 U/ml of Type 1 collagenase (Worthington), 10% FCS, 10 mmol/l HEPES, 1 μmol/l MgCl_2_ and 1 μmol/l CaCl_2_.and incubated at 37 °C for 40 min. Digested tumours were further mechanically disassociated through a 70 μM nylon cell strainer, pelleted, resuspended in 44% isotonic Percoll solution (in HBSS) and then underlaid with 77% isotonic Percoll solution. Cell suspensions were subsequently centrifuged at 2000 rpm for 20 min at room temperature, and cells at the density gradient interface were collected and filtered through a 70 μM nylon cell strainer prior to staining.

Splenocytes and tumour infiltrating cells were stained with fixable viability dye (Zombie Aqua; 1:500; Biolegend), washed in flow cytometry buffer and then treated with anti-mouse CD16/CD32 (Fc Block; 1:300; BD Biosciences), before staining with antibodies targeting the cell surface proteins CD45.2 (104; 1:200), CD8α (53-6.7; 1:200), CD4 (RM4-5; 1:400), CD62L (MEL-14, 1:200), CD44 (IM7; 1:600), PD-1 (29 F.1A12; 1:100), Tim3 (RMT3-23; 1:100), CD11b (M1/70: 1:200), CD11c (N418; 1:200), IA/IE (M5/114.15.2; 1:200; all Biolegend), CD19 (1D3; 1:200) and/or Ly6G (1A8; 1:200; both BD Bioscience). Samples were then fixed and permeabilized using an intracellular transcription factor staining kit (Ebioscience) and stained with anti-FoxP3 (FJK-16s, 1:100; Ebioscience), anti-TCF-7/TCF-1 antibody (S33-966, 1:50; BD Bioscience), anti-Granzyme B (GB12, 1:200; Invitrogen), and/or anti-Ki67 (16A8). Counting beads (CountBright Absolute counting beads; Thermofisher) were added immediately prior to analysis and samples were run on a Cytek Aurora flow cytometer.

### Statistical analyses

GraphPad Prism 10 (v10.2.1) was used for all statistical analyses. Paired or unpaired Student’s *t* test (two-tailed) or one-way ANOVA was used to test significance. Highly skewed data (*p* < 0.05 F-test) was log-transformed prior to calculation. Significance values are stated in all graphs and sample numbers are indicated in figure legends. Numbers of experiments and replicates was based on prior experience of expected biological and technical variability with similar assays, with no data excluded.

## Supplementary information

Supplementary Figure 1

Supplementary Figure 2

Supplementary Figure 3

Supplementary Figure 4

Supplementary Figure 5

Supplementary Figure 6

Supplementary Figure 7

## Data Availability

Data generated as part of this study are available on request from the corresponding authors.
