# Supplementary figures and images for: Dual Bcl-2/Bcl-xl inhibition via AZD0466 combines with immune checkpoint blockade to enhance anti-tumour activity
Source: Cell Death Dis. 2026 Jan 6;17(1):177. doi: 10.1038/s41419-025-08354-w (PMC12876840; doi:10.1038/s41419-025-08354-w)

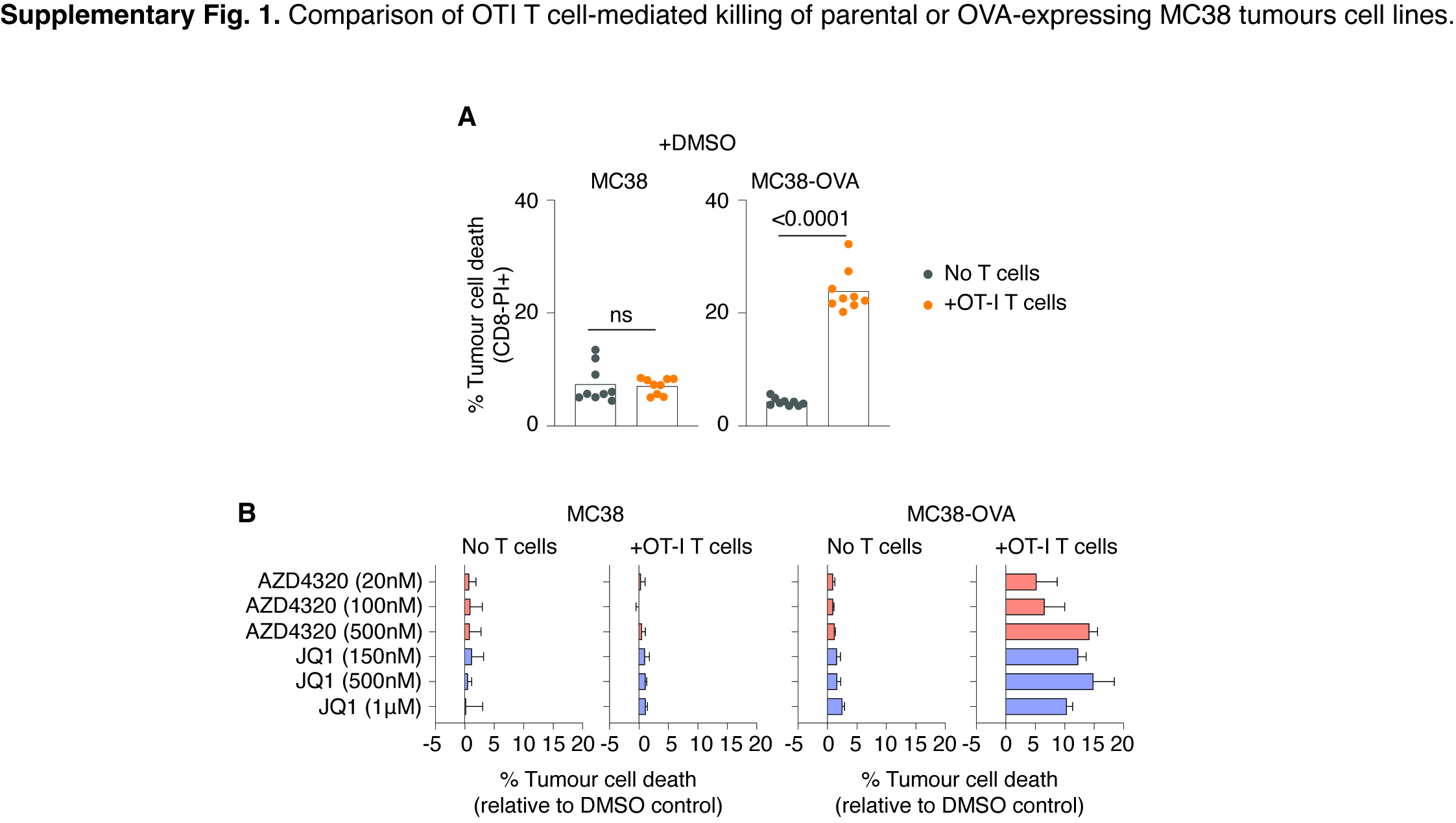

Supplement: Supplementary file 1 — Supplementary Figure 1 [file 41419_2025_8354_MOESM1_ESM.tif]

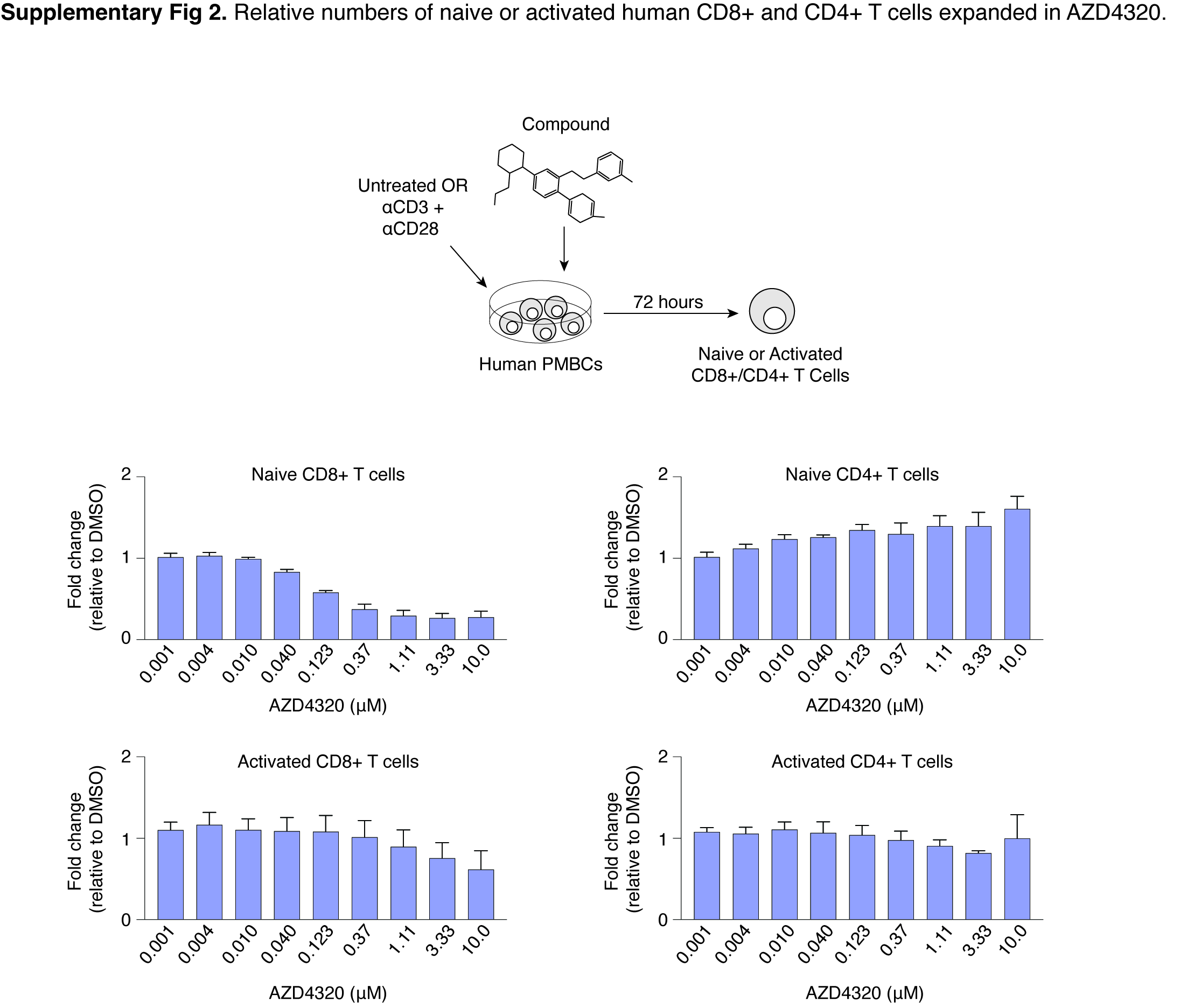

Supplement: Supplementary file 2 — Supplementary Figure 2 [file 41419_2025_8354_MOESM2_ESM.tif]

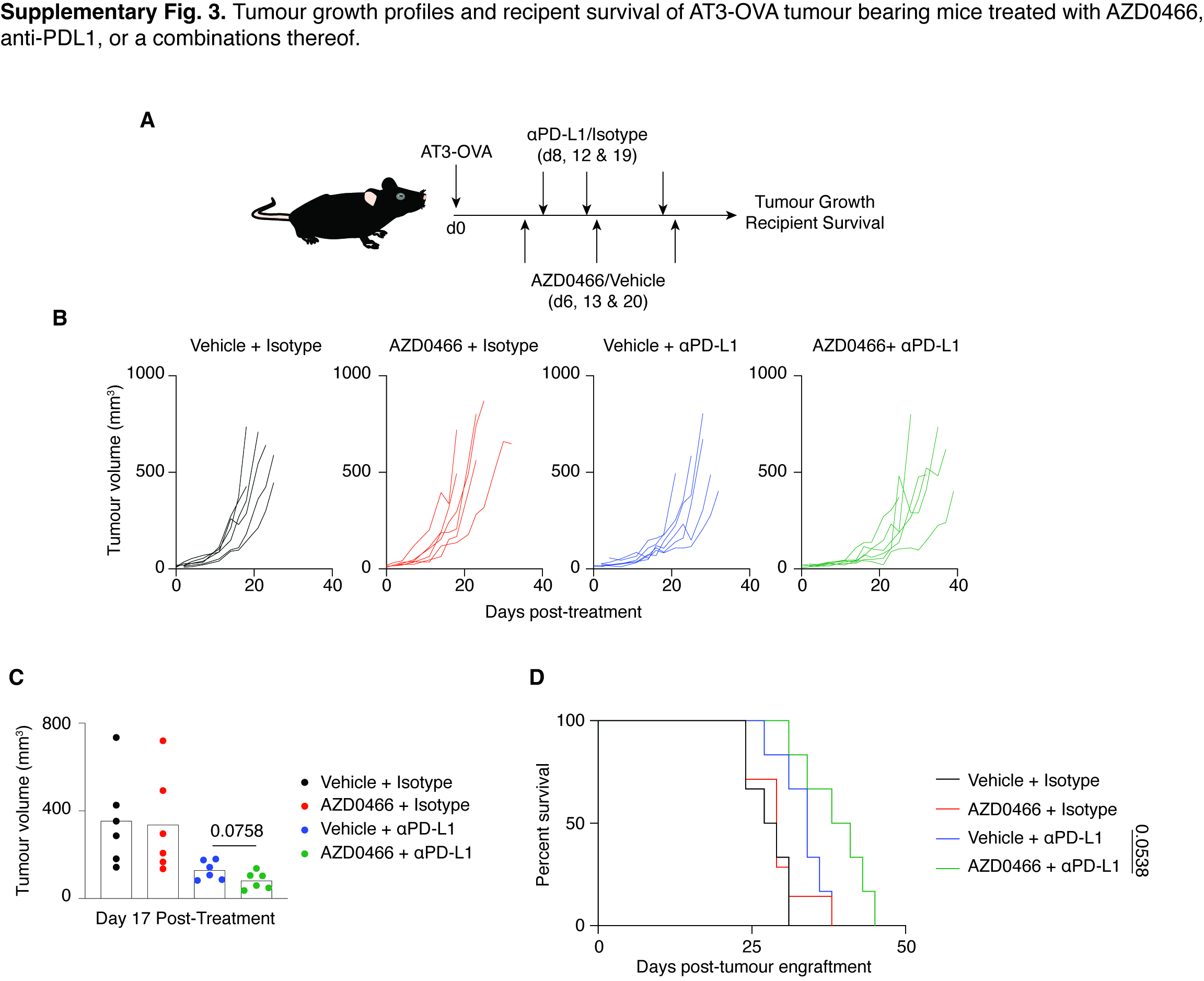

Supplement: Supplementary file 3 — Supplementary Figure 3 [file 41419_2025_8354_MOESM3_ESM.tif]

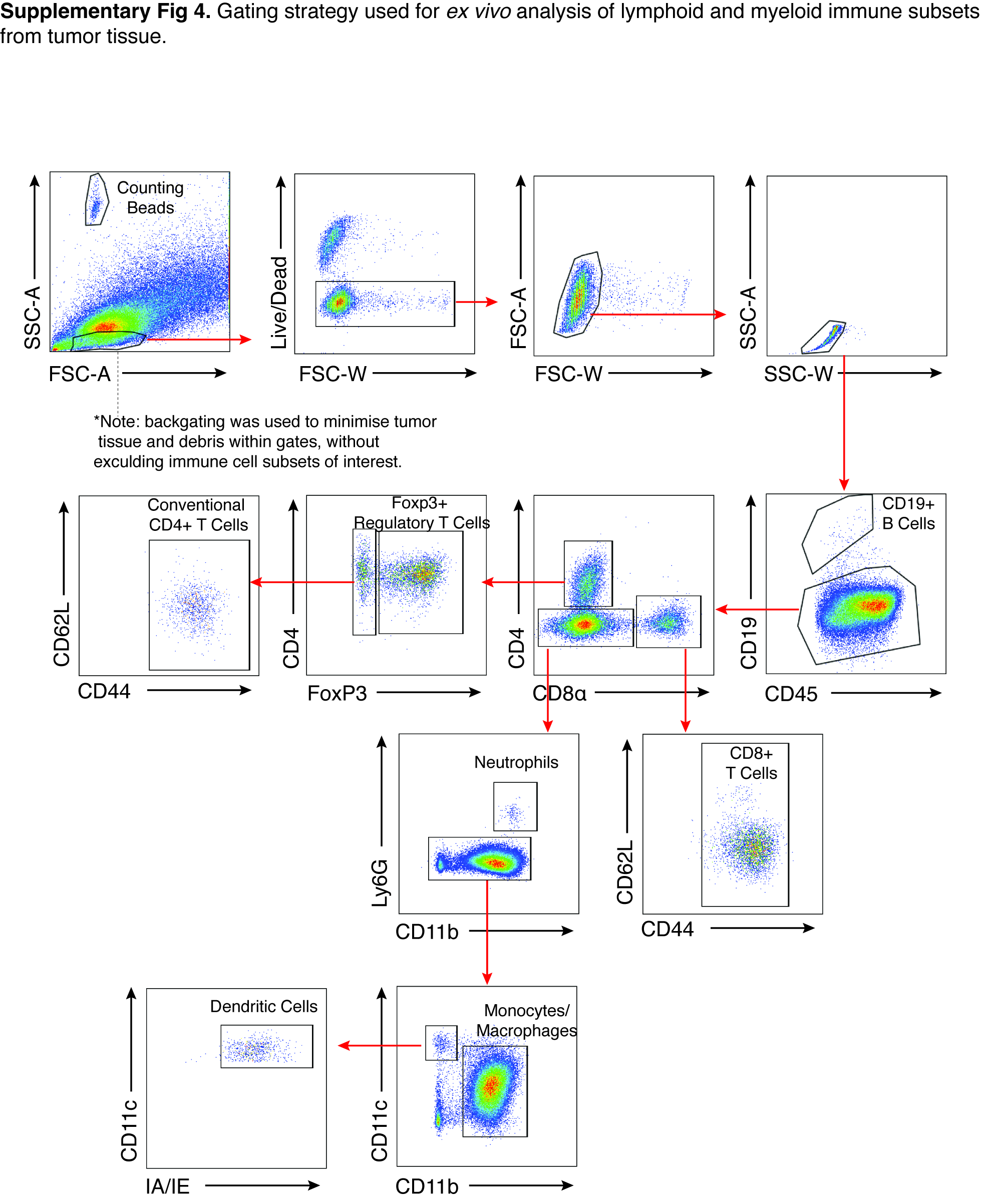

Supplement: Supplementary file 4 — Supplementary Figure 4 [file 41419_2025_8354_MOESM4_ESM.tif]

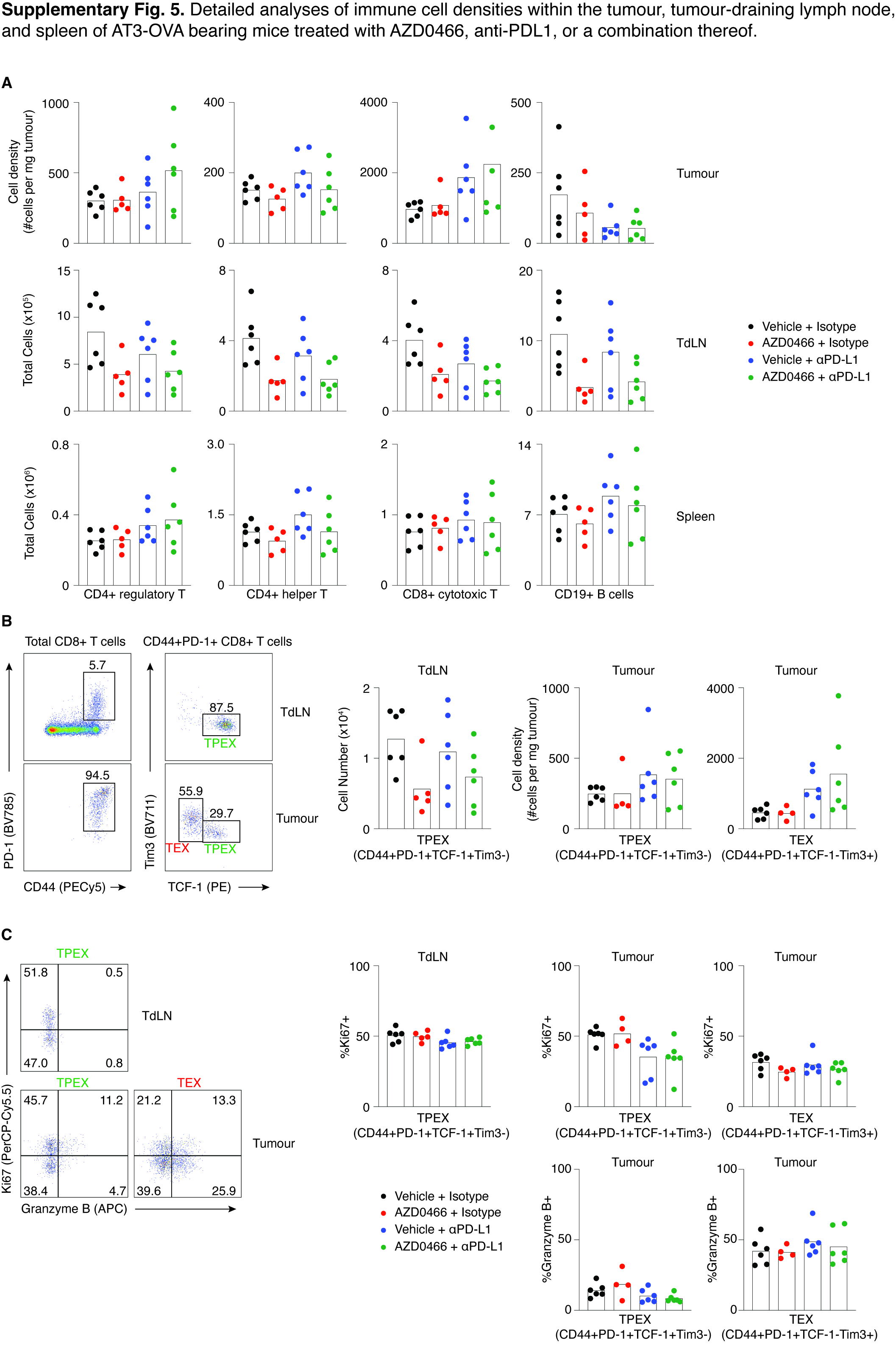

Supplement: Supplementary file 5 — Supplementary Figure 5 [file 41419_2025_8354_MOESM5_ESM.tif]

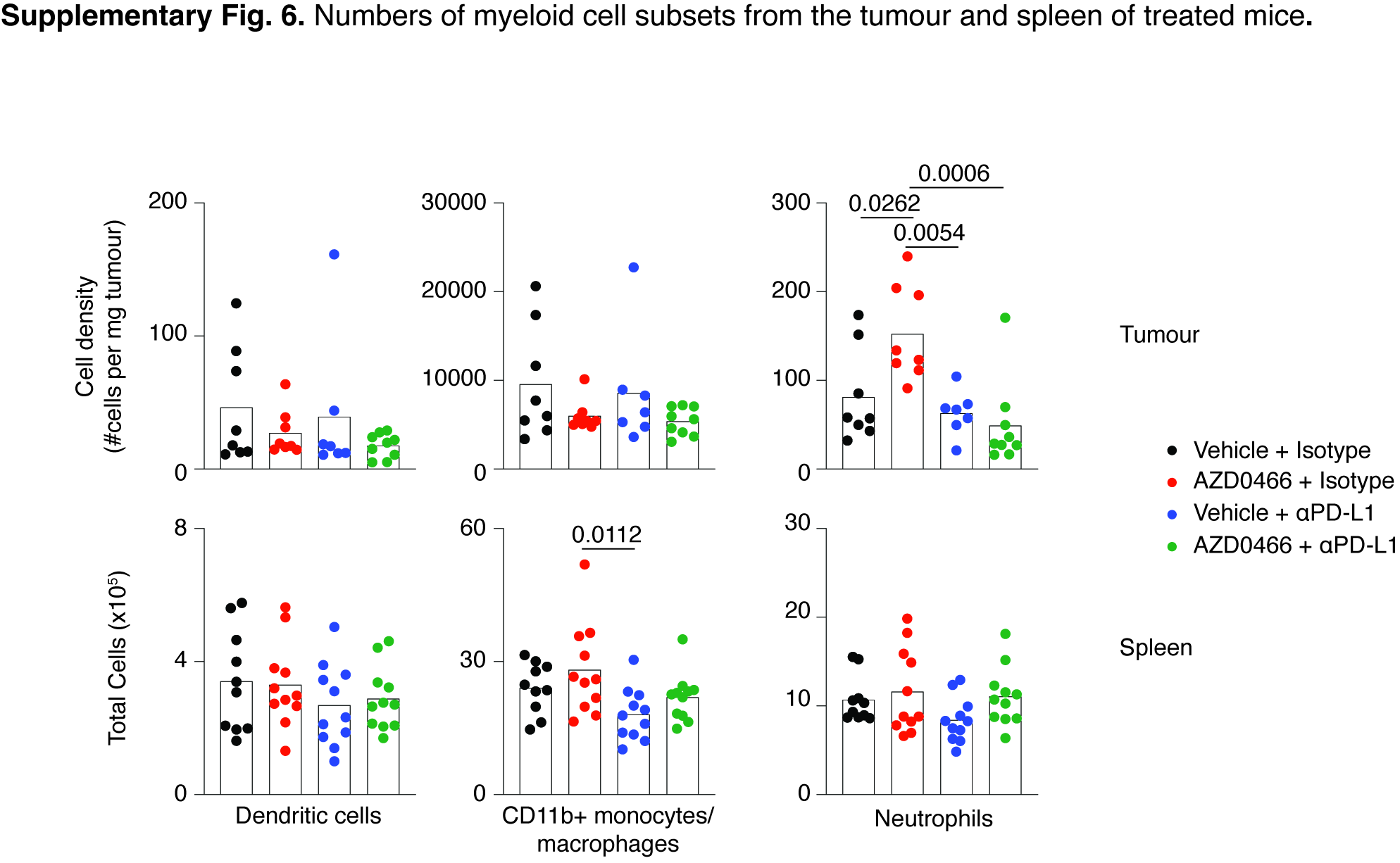

Supplement: Supplementary file 6 — Supplementary Figure 6 [file 41419_2025_8354_MOESM6_ESM.tif]

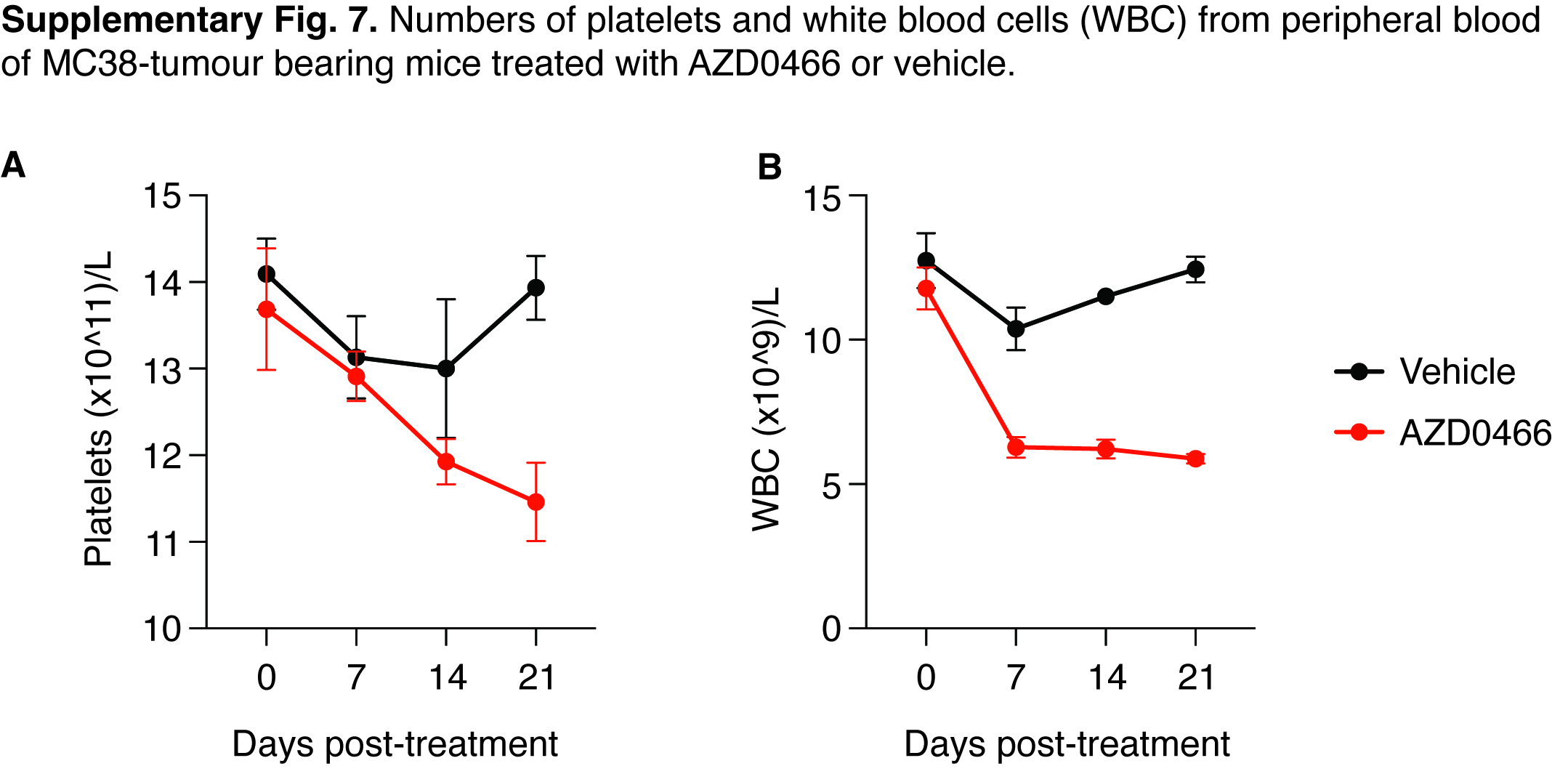

Supplement: Supplementary file 7 — Supplementary Figure 7 [file 41419_2025_8354_MOESM7_ESM.tif]
